# AWOT and CWOT for genotype and genotype-by-treatment interaction joint analysis in pharmacogenetics GWAS

**DOI:** 10.1093/bioinformatics/btac834

**Published:** 2023-01-20

**Authors:** Hong Zhang, Devan V Mehrotra, Judong Shen

**Affiliations:** Biostatistics and Research Decision Sciences, Merck & Co., Inc, Rahway, NJ 07065, USA; Biostatistics and Research Decision Sciences, Merck & Co., Inc, North Wales, PA 19454, USA; Biostatistics and Research Decision Sciences, Merck & Co., Inc, Rahway, NJ 07065, USA

## Abstract

**Motivation:**

Pharmacogenomics (PGx) research holds the promise for detecting association between genetic variants and drug responses in randomized clinical trials, but it is limited by small populations and thus has low power to detect signals. It is critical to increase the power of PGx genome-wide association studies (GWAS) with small sample sizes so that variant–drug-response association discoveries are not limited to common variants with extremely large effect.

**Results:**

In this article, we first discuss the challenges of PGx GWAS studies and then propose the adaptively weighted joint test (AWOT) and Cauchy Weighted jOint Test (CWOT), which are two flexible and robust joint tests of the single nucleotide polymorphism main effect and genotype-by-treatment interaction effect for continuous and binary endpoints. Two analytic procedures are proposed to accurately calculate the joint test *P*-value. We evaluate AWOT and CWOT through extensive simulations under various scenarios. The results show that the proposed AWOT and CWOT control type I error well and outperform existing methods in detecting the most interesting signal patterns in PGx settings (i.e. with strong genotype-by-treatment interaction effects, but weak genotype main effects). We demonstrate the value of AWOT and CWOT by applying them to the PGx GWAS from the Bezlotoxumab *Clostridium difficile* MODIFY I/II Phase 3 trials.

**Availability and implementation:**

The R package COWT is publicly available on CRAN https://cran.r-project.org/web/packages/cwot/index.html.

**Supplementary information:**

Supplementary data are available at *Bioinformatics* online.

## 1 Introduction

Genome-wide association studies (GWAS) provide a hypothesis free approach for identifying associations between genotype and phenotype. To date, GWAS have identified many genetic markers associated with complex diseases and drug responses (Giacomini *et al.*, 2017; McInnes *et al.*, 2021). Pharmacogenomics (PGx) is an important tool for precision medicine, studying how pharmacokinetics, pharmacodynamics, efficacy and safety responses to drugs are associated with genetic information at the molecular level of treated subjects (Chen *et al.*, 2013; Roden *et al.*, 2019). PGx GWAS have been widely used to identify genetic biomarkers associated with drug response and further understand the underly mechanisms of the inter-individual difference in drug responses (Giacomini *et al.*, 2017; McInnes *et al.*, 2021).

In PGx studies, a patient’s clinical outcomes are influenced by both prognostic and predictive factors [see formula (1) in Section 2.2]. A prognostic biomarker, discovered by testing the genotype main effect (G), affects the likelihood of the clinical phenotype regardless of the type of treatment, which is useful in classifying patients into different risk categories indicating the condition of the disease. In contrast, a predictive biomarker, discovered by testing the genotype-by-treatment interaction (GT), affects the likelihood of the clinical event for a treatment, which is useful in segmenting patients into treatment response and nonresponse subgroups. PGx GWAS performed using data from a randomized clinical trial usually must contend with small sample size (i.e. from tens to a few thousand patients), which may severely limit power. Jointly testing the main genetic effect and genotype-by-treatment interaction effect has been shown to increase power for detecting signals in PGx studies compared with only testing the interaction effect or the genotype main effect separately (Kraft *et al.*, 2007).

Traditional methods such as the 2-degree-of-freedom (2df) likelihood ratio test (LRT) or score test are commonly used to assess the combined prognostic and (treatment-related) predictive association of each genetic variant to drug response (Shen *et al.*, 2020). However, these methods have some potential issues when applied to real PGx GWAS data. First, the traditional methods generate inflated (incorrectly small) *P*-values for single nucleotide polymorphisms (SNPs) with low minor allele count (MAC), which may occur due to either small sample size, low minor allele frequency (MAF) or a combination of the two. Second, they also generate inflated (small) *P*-values for binary traits with imbalanced case/control ratios. Generally speaking, inflated type I error (T1E) (or Q–Q plots) in PGx GWAS analysis may be due to three reasons: (i) confounding factors like sample relatedness, population structure, genotyping error or batch effect; (ii) polygenicity, where in truth there are many associated variants (with small effect sizes) and (iii) small sample size and low MAC leading to a deviation of the test statistics from the asymptotic distribution (as described above). In this article, we mainly focus on addressing the third issue.

In addition to adopting the joint test, a second possible way to increase power is to convert the 2df-LRT to a 1df-LRT by using a composite genotype variable constructed from both genotype and genotype-by-treatment interaction and proper weighting between them. Consider as an example the SNP rs2516513 in extended major histocompatibility complex on Chromosome 6, which has been shown to be associated with a reduction of recurrence of *Clostridium difficile* Infection (rCDI) in bezlotoxumab-treated participants (Shen *et al.*, 2020). We can construct a composite genotype variable $Z_{w}=wG+(1-|w|)GT,$ where -1≤w≤1 defines the contribution from the genetic main effect and genotype-by-treatment interaction terms. Considering 21 equal-spaced weights *w *=* *−1.0, −0.9, …, −0.1, 0, 0.1, …, 0.9 and 1, we can see their impact on the converted 1df-LRT *P*-values. Supplementary Figure S1 shows that this strategy does increases power and generates small *P*-values when weights range from −0.3 to 0.4, which essentially puts more weights on the GT interaction term. This is particularly interesting for predictive biomarker detection in the PGx case. Depending on different scenarios or analysis objectives, we can set different types of weights so that they can increase power for detecting signals with a specific signal pattern (i.e. predictive signals with strong GT effects, prognostic signals with strong G effects or both). This simple example motivates us to develop more flexible methods with larger power by converting a 2df test to a 1df test using a composite genotype variable and appropriate weighting.

The third possible way to increase power in certain scenarios is to construct a more robust test which covers more signal patterns. The score test and LRT have different power performance for different signal patterns. LRT under logistic regression tests for the (log) odds ratio, so LRT is more powerful to detect odds ratios. The marginal score test ${\;G}^{T}(Y-\mu^{\left(0\right)})$ essentially models *Y* as a continuous variable and tests its association with *G*. The interpretation of β is that $\beta =E\left(Y=1|G=1\right)-E\left(Y=1|G=0\right)=P\left(Y=1|G=1\right)-P\left(Y=1|G=0\right)=p_{11}-p_{10}=RD$ or risk difference. Thus, the score test targets the RD signals and is more powerful to detect RDs. Signal patterns, whether being large odds ratio (which favors LRT) or large RD (which favors score tests), decide which optimal method should be used for relatively higher power. Combining these two tests creates a more robust test for detecting signals with large effect with either odds ratio or RD.

The objective of this research is to develop more flexible and robust statistical methods for single-variant GWAS analysis with better T1E and larger power than existing methods such as 2df-LRT and 2df-Firth in preferred signal patterns in PGx GWAS. In this article, we adopt the second strategy of converting a 2df test to a 1df test and develop the Adaptively Weighted jOint Test (AWOT) for single-variant 2df tests in PGx GWAS. AWOT first constructs the composite genotype variable using a grid of weights and then uses the minimal *P*-value (minP) approach to find the optimal weight and generate the final *P*-value. We further adopt both the second and third strategies and develop Cauchy Weighted jOint Test (CWOT) for single-variant 2df tests in PGx GWAS. CWOT is an omnibus test that combines the *P*-values from the composite genotype variable based 1df-Score and 1df-LRT methods by using the Cauchy combination test. Our extensive simulations show that the proposed AWOT and CWOT control T1E well and outperform existing methods across preferred signal patterns in PGx GWAS. We further demonstrate the value of AWOT and CWOT by applying them to the PGx GWAS from the Bezlotoxumab *C.difficile* MODIFY I/II Phase 3 trials.

## 2 Materials and methods

### 2.1 Models and existing methods for single-variant analysis in PGx GWAS

We consider a generalized linear model for single-variant analysis in the PGx GWAS scenario

(1) $$g\left(E\left(Y\right)\right)={\;\beta }_{0}+\beta_{T}T+\beta_{X}X+\beta_{G}G+\beta_{GT}GT,$$

where g is the link function, Y is the response, T is the treatment indicator, G is the genotype, and GT is the genotype-by-treatment interaction. We want to test the joint effect:

$$H_{0}:\beta_{G}=\beta_{GT}=0\text{ versus }H_{1}:\beta_{G}\neq 0\;or\;\beta_{GT}\neq 0.$$

Below, we briefly list a few examples of commonly used tests for the above hypothesis testing problem, which will be used to compare with the proposed AWOT and CWOT methods.

1. LRT (or 2df-LRT): LRT (for either continuous or binary trait), $2\left(l_{0}-l_{1}\right)∼\chi_{2}^{2},$ where $l_{i}$ is the maximized log likelihood under $H_{i},\;i=0,\;1.$
2. FT (or 2df-FT): F test (for continuous trait), $\frac{\left(n-p)(\mathrm{RS}S_{0}-\mathrm{RS}S_{1}\right)}{2RSS_{1}}∼F(2,\;n-p)$ where $RSS_{i}$ is the residual sum of squares under $H_{i},\;i=0,\;1$, n is the sample size and p is the number of variables of interests.
3. Firth (or 2df-Firth): Firth’s penalized LRT (Firth, 1993, for binary trait), $2\left({\tilde{l}}_{0}-{\tilde{l}}_{1}\right)∼\chi_{2}^{2},$ where $\tilde{l}\;$is the maximized penalized likelihood with penalty function $\frac{1}{2}l\mathrm{og}\det(I)$, and I is the Fisher’s information matrix, det⁡(I) is the determinant of I. Firth’s method corrects the potential bias of maximum likelihood estimation to improve inference in small samples.

### 2.2 Weighted joint test

Consider the same generalized linear model specified in formula (1) in the PGx scenario. Let $\hat{\mu }$ be the estimator of $E\left(Y\right)$ under the null model. Define the residuals $u=Y-\hat{\mu }$. Define composite variable $Z_{w}=wG+(1-|w|)GT,-1\leq w\leq 1.$ Then, we can define the marginal (1df) score statistic ${S_{w}=Z}_{w}^{\prime }u$. Define composite variable $Z_{w}=wG+(1-|w|)GT,-1\leq w\leq 1.$ Then, we can define the marginal (1df) score statistic ${S_{w}=Z}_{w}^{\prime }u$. The null distribution of $S_{w}$ can be approximated using a normal distribution for continuous traits or saddle point approximation (Dey *et al.*, 2017) for binary traits. We can also conduct a 1df LRT based on $Z_{w}$: $2\left(l_{0}-l_{1}\right)∼\chi_{1}^{2},$ where $l_{0}$ is the maximized likelihood under $H_{0}$, and $l_{1}$ is the maximized likelihood under $H_{1}:g\left(E\left(Y\right)\right)={\;\beta }_{0}+\beta_{T}T+\beta_{X}X+\beta Z_{w}$.

To understand the motivation of the weighted joint test, we can rewrite Equation (1) as follows.

$$\begin{array}{l} g\left(E\left(Y\right)\right)=\beta_{0}+\beta_{T}T+\beta_{X}X+\beta_{G}G+\beta_{GT}G\\ =\beta_{0}+\beta_{T}T+\beta_{X}X\\ +(|\beta_{G}|+|\beta_{GT}|)(\frac{\beta_{G}}{\left|\beta_{G}|+|\beta_{GT}\right|}G+\frac{\beta_{GT}}{\left|\beta_{G}|+|\beta_{GT}\right|}GT)\\ =\beta_{0}+\beta_{T}T+\beta_{X}X+\beta \;\left(w_{G}G+w_{GT}GT\right), \end{array}$$

where $\beta ={|\beta }_{G}|+{|\beta }_{GT}|$ and $w_{G},\;w_{GT}\in \left(-1,\;1\right),\;\left|w_{G}\right|+\left|w_{GT}\right|\equiv 1$.

In light of this transformation, the weighted score test is the score test of a single variable $Z_{w}=\;w_{G}G+w_{GT}GT$ that combines the main term and the interaction term according to some weight. The relationship between the genotype main weight $w_{G}\;$and the interaction weight $w_{GT}$ is illustrated in Supplementary Figure S2a. We can calculate the absolute allele frequency of the composite variable as $|EZ_{w}|/2=|w_{G}+pw_{GT}|\mathrm{maf},\;$where p is the proportion of patients who receive the treatment and maf is the minor allele frequency of the SNP. It is straightforward to check that this function is not symmetric around $w_{G}=0$ as also evidenced by Supplementary Figure S2b. An advantage of this approach is that we can subjectively focus on the effect pattern(s) that we are most interested in by specifying the corresponding weight(s). For example, if we want to discover SNPs that have large interaction effects, then we may define $w_{GT}$ closer to 1 and $w_{G}$ closer to 0. Of course, the optimal weight depends on the ‘balance’ between the true main and the true interaction effect, which is typically unknown. Therefore, we develop an adaptive way to approximate the optimal weight.

### 2.3 Adaptively Weighted jOint Test

To make the score test more robust against unbalanced effect sizes from the main G effect and the genotype-by-treatment interaction effect, we propose to adaptively select the optimal w by grid search. For example, let $w_{1}=w=0,\;0.1,\;0.2,\ldots 1$ and $w_{2}=1-w$ if G and GT have the same direction of effects. Let $w_{1}=w=-1,-0.9,\;\ldots 0,\;0.1,\ldots ,1$ and $w_{2}=1-\left|w\right|$ if they may have different directions of effects. Let $p_{w}$ be the individual *P*-value of $S_{w}={(w}_{1}G+w_{2}GT)'u$. Then, the adaptive score test statistic is $\min P=\underset{w}{\min}p_{w}$.

The *P*-value of min*P* can be calculated by noting that $P\left(\min P>p\right)=P(S_{w}<F_{w}^{-1}\left(p\right),\;w=w_{1},\ldots ,w_{d})$, where $F_{w}^{-1}\left(p\right)$ is the inverse cumulative distribution function of $S_{w},\;w=w_{1},\ldots ,w_{d}$, approximately follows a multivariate normal distribution with mean zero and covariance matrix: $\Sigma_{ij}=Z_{w_{i}}^{\prime }C\mathrm{ov}(u)Z_{w_{j}}^{\prime }$.

### 2.4 Omnibus test—CWOT

As previously noted, the LRT has a different power performance pattern while compared with the score test (Ma *et al.*, 2013). To cover more signal patterns and create a more robust test, we consider extending AWOT to also include the 1df-LRT. The minP test, however, is difficult to be extended to include the 1df-LRT since the joint distribution of score and LRT is unknown. We therefore use the Cauchy *P*-value combination method (Liu and Xie, 2020) to calculate the combined *P*-value

$$T=\sum_{i=1}^{d}\tan\left(\pi \left(0.5-p_{i}\right)\right)/d,$$

where $p_{i},\;i=1,\ldots ,d$ are *P*-values of 1df-LRT and score tests based on different weights. Then,

$$P\left(T>t\right)\approx P\left(W>t\right)=\frac{1}{2}-\frac{\tan^{-1}\left(t\right)}{\pi },$$

for large t, where W is standard Cauchy random variable.

For selecting candidate weights in AWOT or CWOT, if no prior information or subjective preference is available, equal space weights, e.g. w=-1,-0.9, …0, 0.1,…,1 can be used. Although the equal space weights are numerically symmetric around 0, that does not mean they cover the signal patterns symmetrically in terms of interaction/main effect ratio since the absolute allele frequency of the composite genotype is not symmetric around $w_{G}=0$ (Supplementary Fig. S2b). If, however, this information is available, unequal spaced weights are more appropriate. For example, in PGx GWAS, we recommend more dense grids around w=0, since the GT interaction signal is typically rarer than the G term, thus more sensitive to the misspecification of weights. By specifying more weights around w=0, we can also increase the power to detect signals with more interaction effects, which is typically the goal of PGx GWAS.

### 2.5 Simulation study

#### 2.5.1 T1E simulation

We evaluated the T1E and power of the proposed AWOT and CWOT methods through extensive simulations. Under *H*_0_, for continuous traits, we simulated the responses from the following linear model $Y={\;\beta }_{0}+\beta_{T}T+\beta_{X}X$, and, for binary traits from $\mathrm{logit}\left(P\left(Y=1\right)\right)={\;\beta }_{0}+\beta_{T}T+\beta_{X}X$, where a covariate X∼N(0,1), and the treatment variable T∼Bern(0.5). We set the total sample sizes to be 200, 500 and 1000 with treatment-placebo allocation ratio of 1:1. The genotype data G were simulated using the binomial distribution with the probability from MAF, which was set as 0.01 (for sample size = 1000 only), 0.03 (for sample size = 500 and 1000 only), 0.05, 0.15 and 0.25. The intercept ${\;\beta }_{0}$ is set to 0 for continuous traits and −0.75, −1.75 and −2.75 for binary traits. These three intercept values correspond to baseline event rates 32%, 14.8% and 6%, respectively. We applied LRT, FT, AWOT and CWOT_Score to evaluate the T1E rates for continuous traits and LRT, Firth, AWOT and CWOT for binary traits, respectively. CWOT_Score is the Cauchy combination of the weighted score joint test. In total, there are 36 simulation scenarios/combinations of these simulation parameters for binary traits and there are 12 scenarios for continuous traits. Each of the combinations was simulated 10^9^ times except for Firth and AWOT for binary traits, which were simulated 5 × 10^6^ times to conserve computational resources for CWOT and LRT. The empirical T1E rate was calculated as the proportion of *P*-values less than a given level α.

#### 2.5.2 Power simulation

To assess power, responses were generated from the generalized linear model (1). In addition to the scenarios specified in the T1E simulation, we considered effect sizes: $\beta_{G}=\;-2,-1.8,\ldots ,\;2$ and $\beta_{GT}=\;-2,-1.8,\ldots ,\;2$ for continuous endpoints and $\beta_{G}=\;-3,-2,\ldots ,\;3$ and $\beta_{GT}=\;-3,-2.8,\ldots ,\;3$ for binary endpoints. Each of the combinations were simulated 1000 times. The nominal T1E rate was evaluated at a given level α (i.e. $\alpha =5\times 10^{-8}$ and $1\times 10^{-5}$ for continuous traits and $\alpha =5\times 10^{-8}$ for binary traits). The power was calculated as the proportion of calculated *P*-values less than α.

### 2.6 *Clostridium difficile* PGx GWAS data

We applied the proposed AWOT and CWOT methods and the traditional LRT (or 2df-LRT) and Firth (or 2df-Firth) methods to the PGx GWAS data from the MODIFY I (NCT01241552) and MODIFY II (NCT01513239) phase 3 clinical trials (Wilcox *et al.*, 2017). *Clostridium difficile* is the most common cause of infectious diarrhea in hospitalized patients, especially in the developed world. Antibiotic therapy (with metronidazole or oral vancomycin) is usually successful in treating the initial episode of *C.difficile* infection (CDI); however, approximately 15–30% of these patients will have a recurrent episode. While genetic variants among *C.difficile* strains are known to influence virulence and risks for mortality, little is known of the effects of host genetic variations on CDI outcomes (Walker *et al.*, 2013). We are interested in testing whether single genetic variants across the genome were associated with response to bezlotoxumab as defined by a decreased risk of rCDI. An exploratory GWAS investigating whether human genetic variation influences bezlotoxumab response based on this GWAS data and the traditional 2df-LRT method were published before (Shen *et al.*, 2020). The main association analysis in that PGx GWAS was the genotype and genotype-by-treatment interaction joint analysis of drug-induced reduction on rCDI (placebo arm versus bezlotoxumab and bezlotoxumab + actoxumab arms). The details about genotyping, data QC, SNP imputation, covariates and statistical model used in the previous GWAS analysis were introduced in Shen *et al.* (2020) and were followed for the real data analyses in this article.

## 3 Results

### 3.1 Simulation results: T1E

The empirical T1E rates evaluated at $\alpha =1\times 10^{-5}$ and $\alpha =5\times 10^{-8}$ for the joint (or 2df) tests for continuous traits are summarized in Table 1. It shows that the LRT tends to generate inflated T1E while AWOT has much better T1E control (slightly conservative) and the FT controls T1E the best. Supplementary Table S1 and Table 2 summarize the empirical T1E rates evaluated at $\alpha =1\times 10^{-5}$ and $\alpha =5\times 10^{-8}$ for the 2df tests for binary trait, respectively. The event rates P(Y = 1| G, T) corresponding to the intercept${\;\beta }_{0}$ set as −0.75, −1.75 and −2.75 are shown in Supplementary Figure S3. Both tables show that the LRT tends to generate inflated T1E while CWOT controls T1E well (although slightly conservative at $\alpha =5\times 10^{-8}$). The Firth method has the most conservative T1E rate.

**Table 1.** Empirical type I error rates evaluated at $\alpha =5\times 10^{-8}$ and $\alpha =1\times 10^{-5}$ for 2df tests for continuous traits

MAF	*n*	$\alpha =5\times 10^{-8}$				$\alpha =1\times 10^{-5}$			
		CWOT_Score	AWOT	LRT	FT	CWOT_Score	AWOT	LRT	FT
0.05	200	1.07E−08	1.87E−08	**1.44E−07**	4.80E−08	5.64E−06	8.66E−06	**1.86E−05**	9.90E−06
0.15	200	1.60E−08	4.00E−08	**1.72E−07**	6.00E−08	5.87E−06	8.83E−06	**1.89E−05**	1.01E−05
0.25	200	8.00E−09	2.50E−08	**1.91E−07**	4.10E−08	5.99E−06	8.84E−06	**1.88E−05**	1.01E−05
0.03	500	2.01E−08	2.01E−08	**8.97E−08**	5.79E−08	8.34E−06	8.54E−06	**1.31E−05**	1.01E−05
0.05	500	3.20E−08	3.40E−08	**8.60E−08**	4.60E−08	8.42E−06	8.63E−06	**1.31E−05**	1.01E−05
0.15	500	3.10E−08	3.30E−08	**8.11E−08**	5.01E−08	8.31E−06	8.37E−06	**1.31E−05**	1.01E−05
0.25	500	3.00E−08	3.40E−08	**7.90E−08**	4.50E−08	8.34E−06	8.31E−06	**1.31E−05**	1.01E−05
0.01	1000	4.40E−08	4.70E−08	**6.90E−08**	5.70E−08	9.22E−06	9.07E−06	1.14E−05	9.99E−06
0.03	1000	4.90E−08	4.60E−08	**7.30E−08**	5.50E−08	9.08E−06	8.92E−06	1.13E−05	9.89E−06
0.05	1000	3.70E−08	3.80E−08	6.10E−08	5.50E−08	9.22E−06	9.07E−06	1.12E−05	9.86E−06
0.15	1000	5.00E−08	4.70E−08	**7.10E−08**	6.20E−08	9.32E−06	9.09E−06	1.14E−05	9.94E−06
0.25	1000	3.50E−08	4.10E−08	6.40E−08	4.80E−08	9.31E−06	9.11E−06	1.14E−05	1.00E−05

*Note*: $\beta_{T}=0.5$, number of simulations = 10^9^. CWOT_Score, CWOT using only score test *P*-values at each weight; FT, F test. Type I error ≥ 1.3α = $6.5\times 10^{-8}\;$and $1.3\times 10^{-5}$ (3*SE) is marked in bold, respectively.

**Table 2.** Empirical type I error rates evaluated at $\alpha =5\times 10^{-8}$ for 2df tests for binary traits

		${\;\beta }_{0}=-0.75$		${\;\beta }_{0}=-1.75$		${\;\beta }_{0}=-2.75$	
MAF	*n*	CWOT	LRT	CWOT	LRT	CWOT	LRT
0.05	200	3.40E−08	2.90E−08	3.30E−08	3.80E−08	3.80E−08	4.64E−08
0.15	200	5.30E−08	**1.02E−07**	4.60E−08	**6.90E−08**	2.93E−08	6.59E−08
0.25	200	5.78E−08	**7.68E−08**	5.43E−08	**9.57E−08**	2.59E−08	7.97E−08
0.03	500	5.31E−08	**6.50E−08**	4.81E−08	3.94E−08	2.66E−08	1.98E−08
0.05	500	5.56E−08	**7.35E−08**	4.00E−08	3.70E−08	3.23E−08	3.35E−08
0.15	500	5.10E−08	5.70E−08	4.10E−08	7.50E−08	2.73E−08	4.48E−08
0.25	500	4.60E−08	5.80E−08	4.40E−08	6.70E−08	2.98E−08	**6.52E−08**
0.01	1000	2.00E−08	2.10E−08	3.80E−08	3.70E−08	5.00E−08	3.41E−08
0.03	1000	5.60E−08	7.90E−08	5.20E−08	4.60E−08	3.49E−08	2.48E−08
0.05	1000	5.60E−08	6.10E−08	4.10E−08	6.00E−08	3.11E−08	2.45E−08
0.15	1000	5.30E−08	4.80E−08	5.20E−08	5.10E−08	4.36E−08	**8.49E−08**
0.25	1000	5.70E−08	5.30E−08	4.60E−08	6.00E−08	3.89E−08	**6.75E−08**

*Note*: ${\;\beta }_{0}$ is set as −0.75, −1.75 and −2.75, respectively, $\beta_{T}=0.5$, number of simulations = 10^9^. Type I error ≥ 1.3α = $6.5\times 10^{-8}\;$(3*SE) is marked in bold.

### 3.2 Simulation results: power comparison

**Fig. 1.**
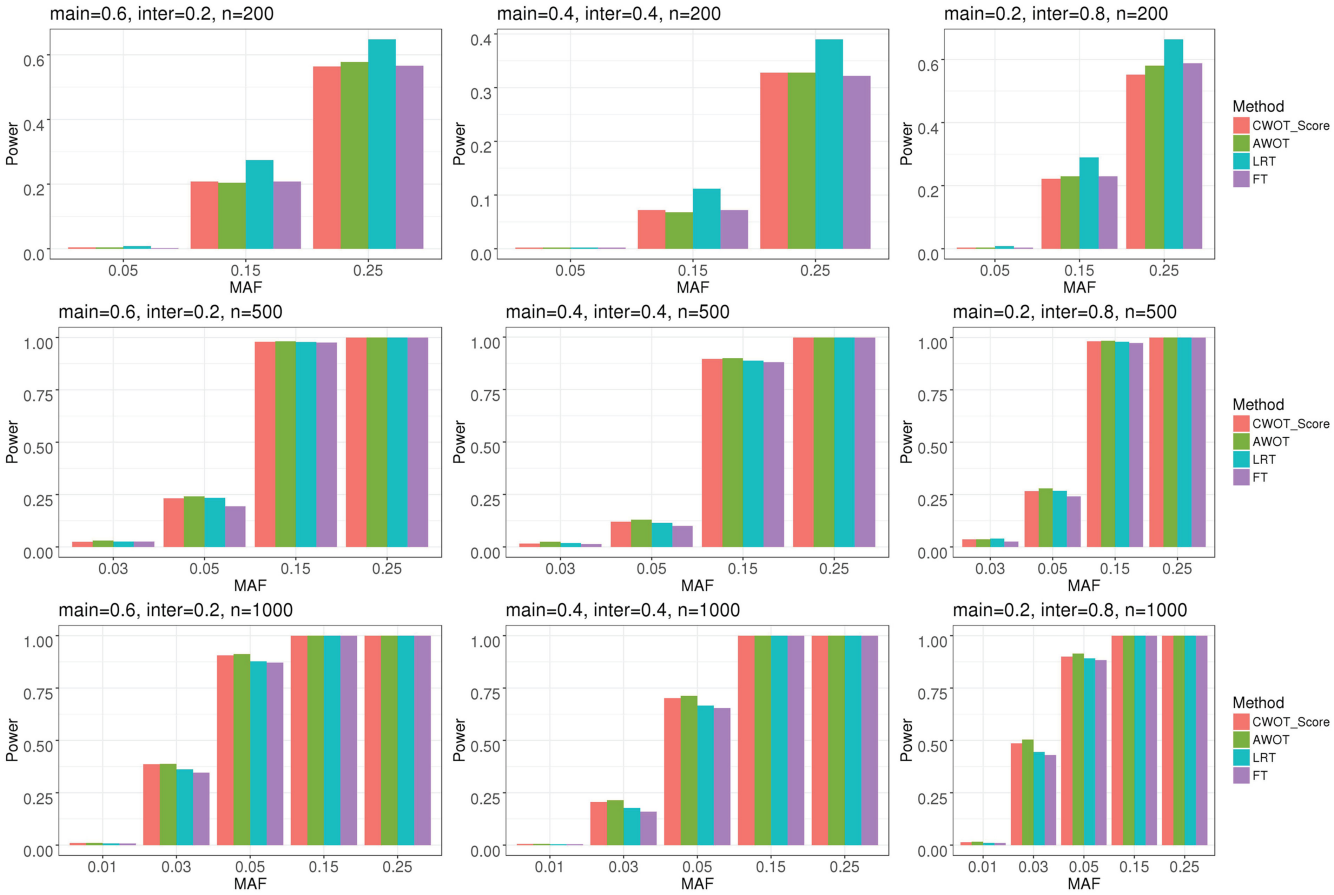
Power comparison of LRT, FT, AWOT and CWOT_Score evaluated at $\alpha =5\times 10^{-8}$ for 2df test of continuous traits. ‘main’ denotes the main G effect size *β_G_*, inter denotes the G*T interaction effect size *β_GT_*, and n is sample size. MAF, minor allele frequency; CWOT_Score, Cauchy combination of the weighted score joint test

**Fig. 2.**
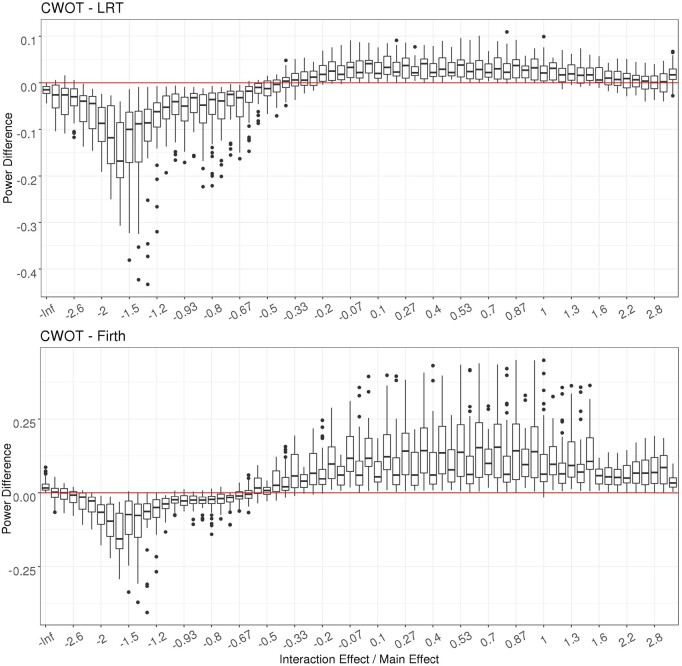
Power difference between CWOT and LRT (upper) and Firth (bottom) evaluated at $\alpha =5\times 10^{-8}$ for 2df test of binary traits. The x-axis is the ratio between the interaction effect *β_GT_* and the main effect *β_G_*. Each boxplot includes all the power differences between CWOT and the competing method when the interaction/main effect ratio is fixed at a certain level

Figure 1 summarizes the power comparison of LRT, FT, AWOT and CWOT_Score (Cauchy combination of the weighted score joint test) evaluated at $\alpha =5\times 10^{-8}$ for 2df tests for continuous traits. It shows that AWOT provides larger power than FT and LRT in all scenarios except for small sample sizes (*n* = 200), which may be due to largely inflated T1E from the LRT test. Note that the power presented in Figure 1 does not adjust the T1E inflation/deflation. The power difference between CWOT, LRT and Firth is summarized in Figure 2, evaluated at $\alpha =5\times 10^{-8}$ for 2df tests of binary traits. The proposed CWOT has higher power than 2df- LRT and 2df-Firth when the main effect and interaction effect are in the same direction, or when the main effect size is at least two times larger than the interaction effect size. CWOT has larger power improvement from 2df-Firth while compared with that from 2df-LRT. The power comparison among 2df-LRT, 2df-FT, AWOT and CWOT for binary traits are summarized in Supplementary Figures S4–S6, corresponding to β_0_ = −0.75, −1.75 and −2.75, respectively. They demonstrate very similar power comparison patterns: CWOT and AWOT outperform LRT and Firth in all settings except when the sample size is small (*n* = 200) and β_0_ is −0.75.

**Fig. 3.**
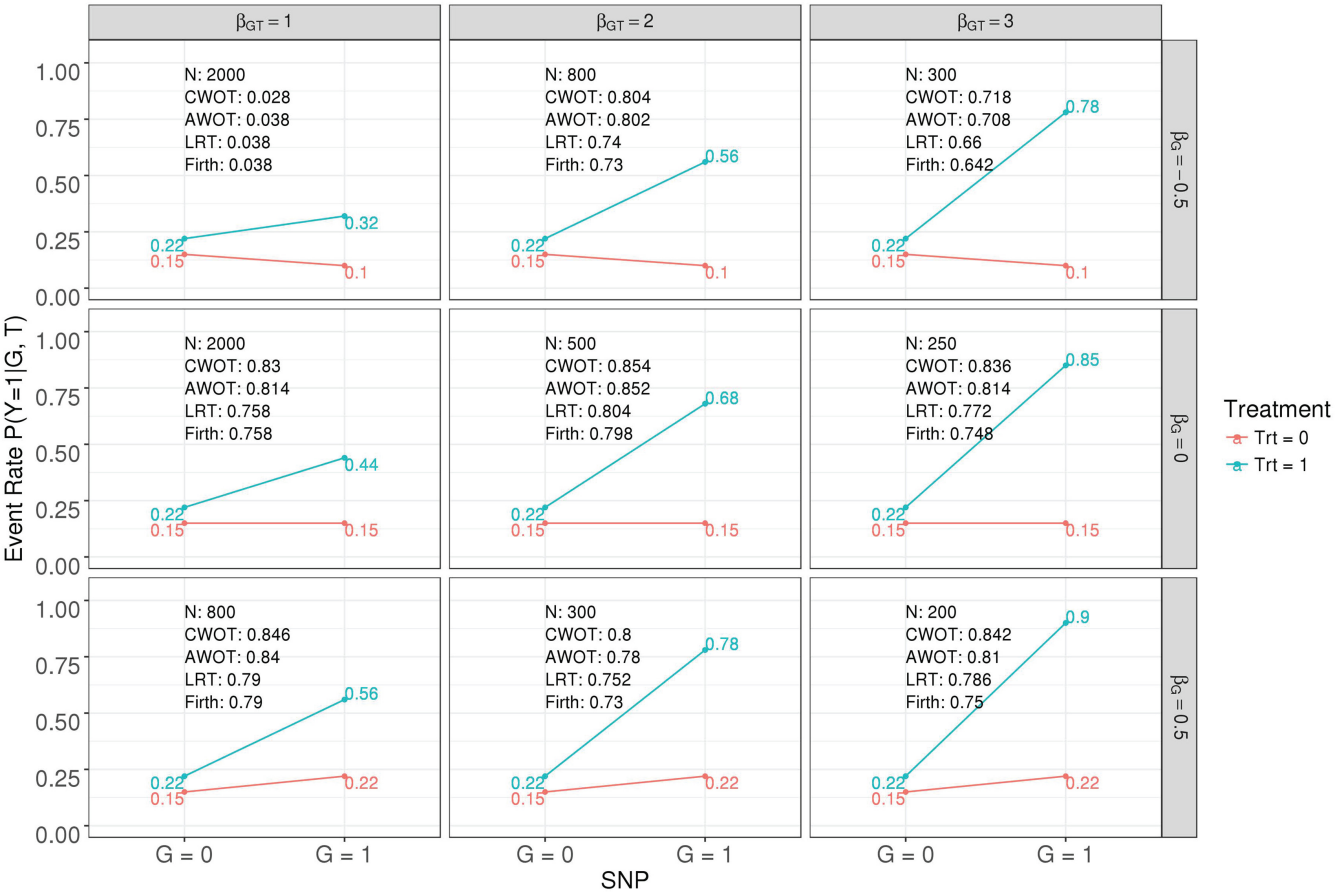
Power comparison among LRT, Firth, AWOT and CWOT evaluated at $\alpha =5\times 10^{-8}$ for 2df test of binary traits. The SNP MAF = 15%, $\beta_{T}$ = 0.5, $\beta_{0}$ = −1.75, sample size is adjusted for proper power. G = 0: SNP−; G = 1: SNP+

In addition, we also compare the power across LRT, Firth, AWOT, and CWOT methods in the most interesting scenarios in PGx settings, e.g. the main effect is weak while the interaction effect is large and in the same direction with the treatment effect. The results are summarized in Figure 3. It further demonstrates that the proposed CWOT method provides larger power in detecting the most interesting PGx signal patterns than 2df LRT and Firth methods.

### 3.3 Analysis of the *C.difficile* PGx GWAS data

We applied the proposed AWOT and CWOT methods and the traditional LRT (or 2df-LRT) and Firth (or 2df-Firth) methods to the GWAS genotype and genotype-by-treatment interaction joint analysis of drug-induced reduction on the recurrence of *C.difficile* infection (rCDI) (placebo arm versus bezlotoxumab and bezlotoxumab + actoxumab arms) in the Phase 3 MODIFY I/II trials. For the CWOT method, we set the weights in three ways (i) equally spaced weights: ±(1, 0.9, 0.8,…,0) (CWOT_ESW); (ii) unequally spaced weights putting more weights around 0 and 1 (CWOT_uESW01): the weights were defined by a piecewise function ${f(x)=-1+4(0.1x)}^{3},\;x=0,1,\;\ldots ,\;4;{f(x)=4(0.1x-1)}^{3},\;x=5,\;6,\;\ldots ,\;15;\;{f(x)=1+4(0.1x-2)}^{3},\;x=16,\;17,\;\ldots ,\;20$, i.e. ±(1, 0.996, 0.968, 0.892, 0.744, 0.5, 0.256, 0.108, 0.032, 0.004, 0); (iii) unequally spaced weights putting more weights around 0 only (CWOT_uESW0): ${f(x)=(0.1x-1)}^{3},\;x=0,\;1,\;\ldots ,\;20$, i.e. ±(1, 0.729, 0.512, 0.343, 0.216, 0.125, 0.064, 0.027, 0.008, 0.001, 0).

**Fig. 4.**
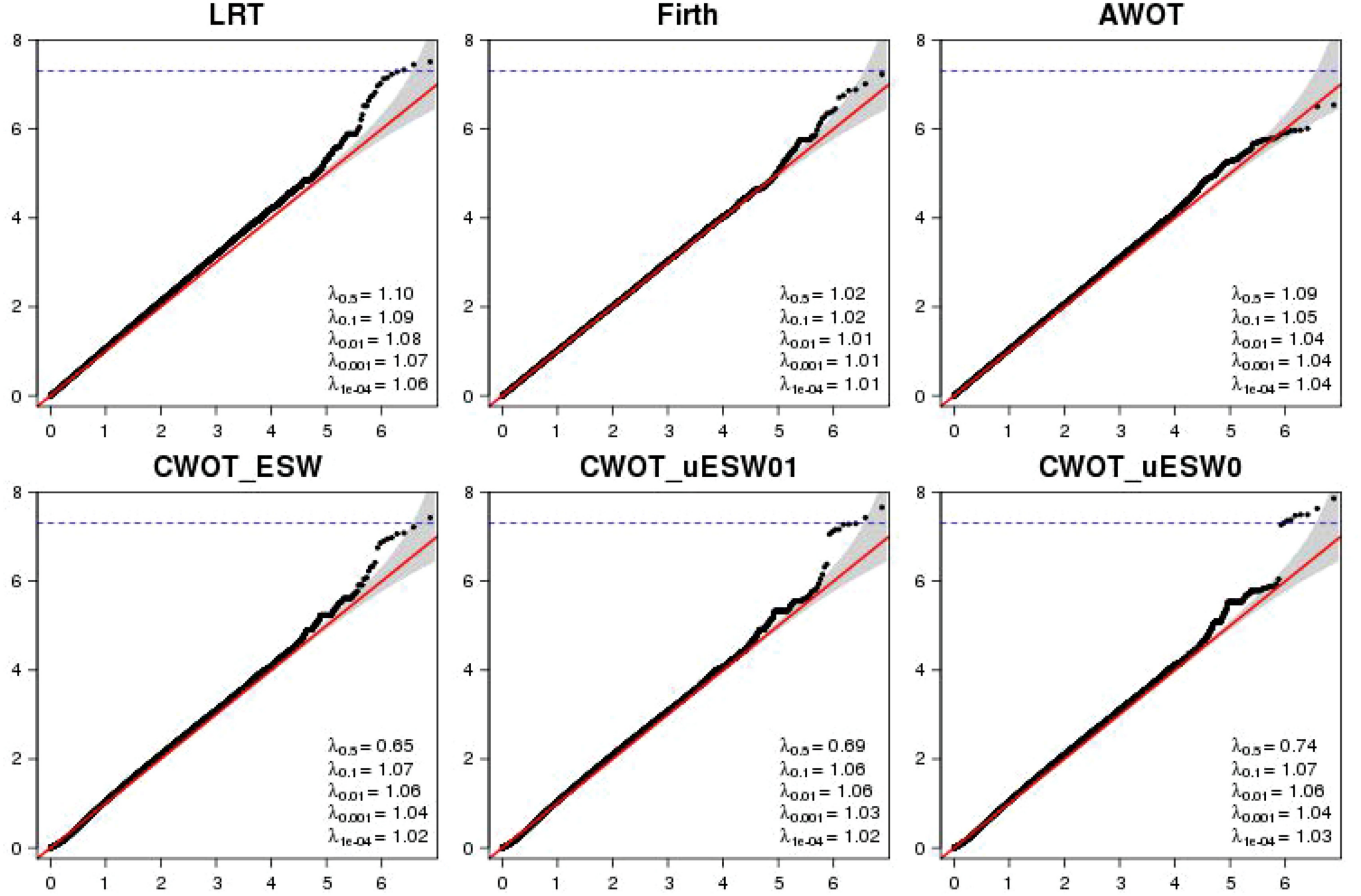
QQ plots of GWAS genotype and genotype-by-treatment interaction joint analysis of drug-induced reduction on rCDI (placebo arm versus bezlotoxumab and bezlotoxumab + actoxumab arms) from the LRT, Firth, AWOT and CWOT_ESW, CWOT_uESW01 and CWOT_uESW0 methods. The dotted blue line is the genome-wide significance *P-*value threshold of 5 × 10^−8^. *λ_q_*, genomic inflation factor at the *q*th *P*-value quantiles. GWAS, genome-wide association study; rCDI, recurrent *Clostridium difficile* infection; SNP, single nucleotide polymorphism. CWOT_ESW (equally spaced weights: −1, −0.9, …, 0.9, 1). CWOT_uESW01 (weight more around 0, 1). CWOT_uESW0 (weight more around 0 only)

The QQ plots of the *P*-values from these methods are summarized in Figure 4 and the Manhattan plots in Supplementary Figure S8. The genome inflation factors λ_0.5_ for the three CWOT methods are considerably <1, which is expected since the Cauchy Combination Test (Liu and Xie, 2020) step is adopted to combine *P*-values. Supplementary Figure S9 compares the *P*-values between Firth, AWOT, CWOT_ESW, CWOT_uESW01 and CWOT_uESW0 and LRT. From these figures, we can see that CWOT generally controls T1E better than LRT and is flexible in providing larger power to detect interesting signal patterns in the PGx setting (i.e. with strong genotype-by-treatment interaction effects, but weak genotype main effect). CWOT_uESW0 is similar as testing the G*T interaction only, but with much larger power since it uses the joint test and optimal weight searching. This method is good for PGx GWAS analysis to detect the predictive genetic biomarkers associated with drug response. CWOT_uESW01 searches both prognostic and predictive biomarkers, with larger power than LRT in some scenarios. Table 3 summarizes the association results of the GWAS genotype and genotype-by-treatment interaction joint analysis of drug-induced reduction on rCDI (placebo arm versus bezlotoxumab and bezlotoxumab + actoxumab arms) from the LRT, Firth, AWOT and CWOT_ESW, CWOT_uESW01 and CWOT_uESW0 methods. LRT detected three SNPs while CWOT_uESW0 detected eight SNPs. The *P*-values from CWOT_uESW0 were generally smaller than those from LRT. The optimal weights which yielded the minimal *P*-value for each of the AWOT and CWOT_ESW, CWOT_uESW01 and CWOT_uESW0 methods are further provided in the Supplementary Table S2. All the optimal weights were small around zero, which indicates that the joint tests were mainly driven by the genotype-by-treatment interaction effects (i.e. what we were looking for). In summary, CWOT provides a more flexible and robust alternative method with better T1E and power performance for PGx GWAS data analysis compared with existing methods.

**Table 3.** Association results of the GWAS genotype and genotype-by-treatment interaction joint analysis of drug-induced reduction on rCDI (placebo arm versus bezlotoxumab and bezlotoxumab + actoxumab arms) from the LRT, Firth, AWOT and CWOT_ESW, CWOT_uESW01 and CWOT_uESW0 methods

SNP	CHR	POS	MAF	*β_G_*	*β_G_* SE	*β_GT_*	*β_GT_* SE	LRT.p	Firth.p	AWOT.p	CWOT_ ESW.p	CWOT_uESW01.p	CWOT_uESW0.p
rs2516513	6	31447588	0.23	−0.01	0.22	−0.88	0.23	**3.04E−08**	5.84E−08	9.70E−7	**3.71E−08**	**2.18E−08**	**1.37E−08**
rs2516509	6	31449994	0.24	−0.02	0.22	−0.83	0.22	5.14E−08	9.62E−08	1.06E−06	6.03E−08	**3.67E−08**	**2.31E−08**
rs113379306	6	17333351	0.04	1.67	0.52	−17.51	636.46	**3.54E−08**	4.44E−07	6.24E−05	1.05E−07	5.21E−08	**3.17E−08**
rs2523705	6	31451680	0.23	−0.02	0.22	−0.83	0.22	7.02E−08	1.30E−07	1.29E−06	8.23E−08	5.03E−08	**3.18E−08**
rs2248462	6	31446796	0.23	−0.03	0.22	−0.82	0.22	7.33E−08	1.36E−07	1.42E−06	8.58E−08	5.28E−08	**3.33E−08**
rs76166871	6	17329940	0.04	1.67	0.52	−17.53	645.60	**4.64E−08**	5.74E−07	5.69E−05	1.38E−07	6.88E−08	**4.19E−08**
rs2516511	6	31448625	0.24	−0.01	0.22	−0.82	0.22	9.49E−08	1.75E−07	2.01E−06	1.12E−07	6.83E−08	**4.31E−08**
rs2516422	6	31449269	0.23	−0.02	0.22	−0.82	0.22	1.07E−07	1.96E−07	1.65E−06	1.25E−07	7.73E−08	**4.88E−08**

*Note*: The *P*-values passing the genome-wide significance threshold 5 × 10^−8^ are highlighted in bold. ‘.p’, *P*-value; SNP, single nucleotide polymorphism; CHR, chromosome; POS, position; MAF, minor allele frequency.

## 4 Discussion

To date, many associations between genetic variation and inter-individual difference in drug response have been discovered to tailor treatments to the genetic makeup of the patient for patient stratification and therapeutic value propositions (Nelson *et al.*, 2016). However, PGx GWAS face unique challenges including small sample size, limited global representation and traditional statistical tests with low power (Bienfait *et al.*, 2022; McInnes *et al.*, 2021). To detect genetic biomarkers that can explain more (unexplained) heritability in drug response, the field needs larger sample sizes, more diverse cohorts and a broader array of statistical tests (McInnes *et al.*, 2021). In this article, we focus on addressing the challenges from the statistical methods or tests perspective.

Motivated by the fact that most of current PGx GWAS with small sample sizes tend to be under-powered for detecting signals with moderate or small effect sizes, we develop two flexible and robust methods AWOT and CWOT for single-variant joint analysis of genotype and genotype-by-treatment interaction in PGx GWAS. Compared with traditional 2df-LRT and 2df-score tests, AWOT and CWOT are designed to flexibly capture different (or imbalanced) relationships between the genetic main (or prognostic) effect and genotype-by-treatment (or treatment-related predictive) effect for each genetic variant and provide larger power in most scenarios, particularly in preferred scenarios from the PGx setting. The flexibility of the tests mainly comes from the strategy of converting the 2df test to a 1df test using a composite genotype variable and by proper weighting, which increases the power as well. Given the fact that the Score test and LRT have different power performance for different binary signal patterns (i.e. the LRT is more powerful to detect odds ratios and the Score test is more powerful to detect RDs), our omnibus test CWOT that combines the 1df-Score and 1df-LRT yields better T1E control and larger and more robust power performance in preferred scenarios in PGx GWAS when compared to the 2df-LRT.

Regarding 2df test for continuous traits, 2df-LRT tends to generate inflated T1E when sample size is small, or when SNPs have small MAF or low MAC. AWOT has much better T1E control (slightly conservative) in both our simulation studies and real data analyses. AWOT has larger power than FT in all scenarios and LRT in all scenarios except for small sample size (*N* = 200), which may be due to the largely inflated T1E from LRT test. CWOT_Score is the Cauchy combination of the weighted score joint test, AWOT is a minP and multi-variant normal approximation-based method. Thus, CWOT-Score is computationally more efficient compared with AWOT. In addition, CWOT-Score and AWOT perform similar in terms of T1E control (both are slightly conservative) and power (AWOT provides slightly larger power compared with CWOT-Score in most scenarios). AWOT is preferred for the analysis of continuous traits unless computational cost is an issue. In PGx GWAS, the sample sizes are usually small, and the computational cost may not be an issue, so we recommend using AWOT rather than CWOT-score for the joint effects test for continuous traits in PGx GWAS. Regarding 2df test for binary traits, LRT tends to generate inflated T1E while CWOT controls T1E well (slightly conservative). Firth method has the most conservative T1E rate. The proposed CWOT has higher power than 2df-LRT when the main effect and interaction effect are not in different directions, or when main effect size is roughly at least double the interaction effect size. We thus recommend using CWOT for the joint effects test with binary traits in PGx GWAS. As a practical guidance, we recommend using AWOT for joint effects test for continuous traits and CWOT for joint effects test for binary traits in PGx GWAS.

In AWOT and CWOT, setting appropriate weights is critical for detecting different types of signals. For example, putting more weights around 0 tends to detect predictive biomarkers more easily with strong genotype-by-treatment interaction effect; putting more weights around 1 tends to detect prognostic biomarkers more easily with strong genotype main effect while putting more weights around both 0 and 1 tends to detect both prognostic and predictive biomarkers more easily. Depending on a specific analysis goal, there is flexibility to use different weights for GWAS analysis. It is good to conduct additional sensitivity analyses using different weight assignments and investigating their impact on signal detection. Applying AWOT/CWOT to PGx GWAS can be regarded as a good screening step for detecting signals which combines both the predictive (or interaction) effect and the prognostic (or the main G) effect. After the top signals are obtained, additional analyses can be conducted to further tease out the predictive (or interaction) effect and the prognostic (or the main G) effect by using any other traditional methods. For example, if one is interested in getting the G*T interaction test only results while conditional on G term in the model, any other traditional methods introduced in Kraft *et al.* (2007) can be used. A computational time comparison (Supplementary Fig. S7) shows that although AWOT and CWOT require more computational time than existing methods such as the LRT and FT, they are still computationally feasible for PGx GWAS analysis with moderately large sample size. We developed an R package ‘CWOT’ for wide use of our proposed methods, which is available on CRAN. We envision that AWOT and CWOT will increase the efficiency for discovering genetic biomarkers associated with drug responses in PGx GWAS for the development of precision medicines.

## Supplementary Material

btac834_Supplementary_Data

## Data Availability

MSD’s data sharing policy, including restrictions, is available at http://engagezone.msd.com/ds_documentation.php. Requests for access to the *C. difficile* PGx GWAS data can be submitted through the EngageZone site or via email to dataaccess@merck.com.
