# Supplementary material for: AWOT and CWOT for genotype and genotype-by-treatment interaction joint analysis in pharmacogenetics GWAS
Source: Bioinformatics. 2023 Jan 20;39(1):btac834. doi: 10.1093/bioinformatics/btac834 (PMC9885423; doi:10.1093/bioinformatics/btac834)
Supplement: btac834_Supplementary_Data [file btac834_supplementary_data.pdf]

Supplementary Materials:

AWOT and CWOT for genotype and genotype by treatment interaction joint analysis in pharmacogenetics GWAS

Hong Zhang[1,3], Devan V. Mehrotra[2] and Judong Shen[1]*

[1]Biostatistics and Research Decision Sciences, Merck & Co., Inc., Rahway, NJ 07065 USA
[2]Biostatistics and Research Decision Sciences, Merck & Co., Inc., North Wales, PA 19454 USA
[3]Present address: Global Biometrics & Data Management, Pfizer Inc., Cambridge, MA 02139 USA

*Corresponding author: judong.shen@merck.com

Supplementary Figures and Tables

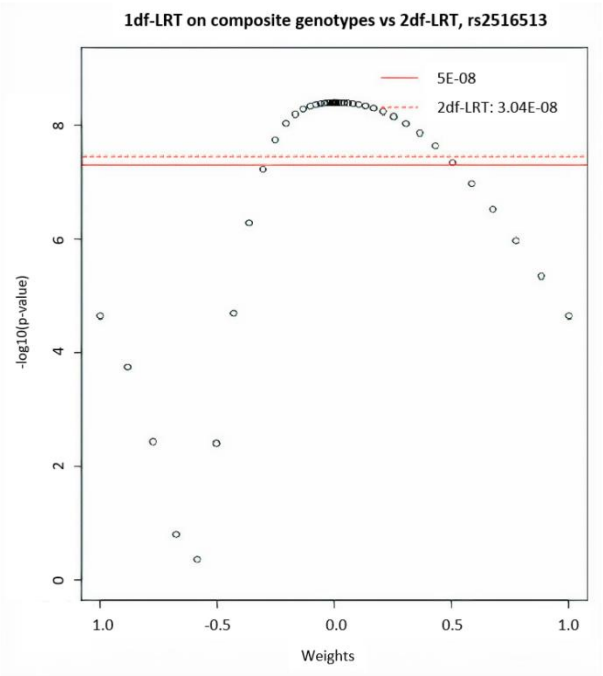

Figure S1. P-value comparison between 2df-LRT and 1df-LRT based on composite variable with proper weighting. A composite genotype variable is defined as $Z_w = wG + (1 - |w|)GT,$ where $-1 \leq w \leq 1.$ 21 weights are considered here: w = -1.0, -0.9, …, -0.1, 0, 0.1, …, 0.9 and 1. The SNP and the clinical data are from Shen et al., 2020.

a)

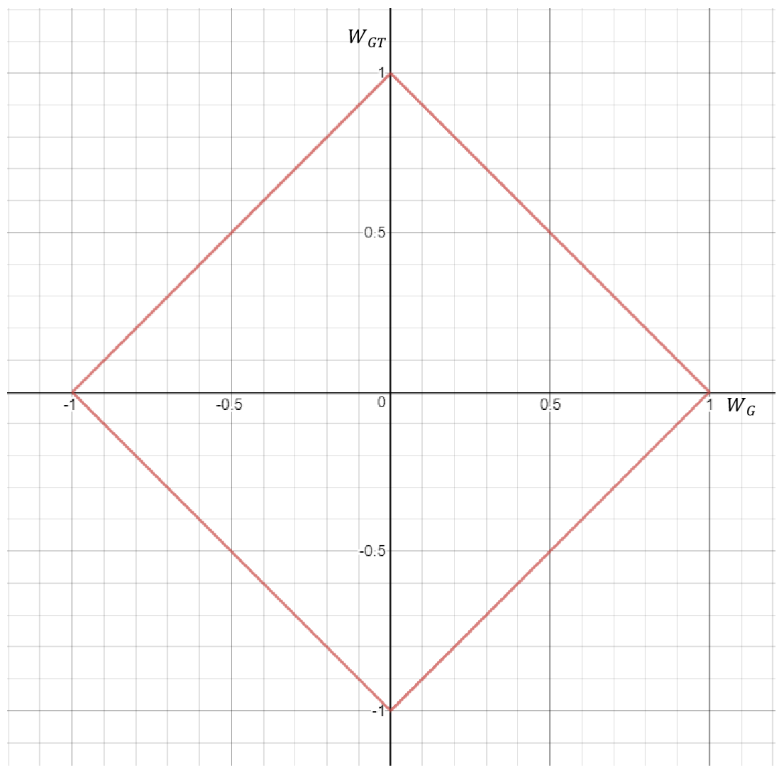

b)

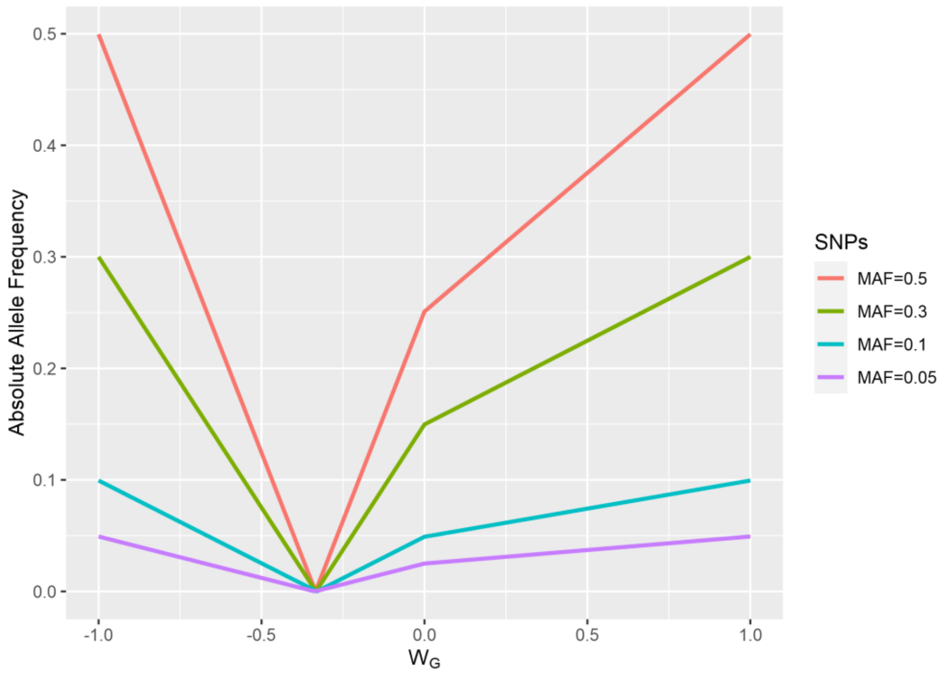

Figure S2. a) The relationship between the main genotype weight w_G and the interaction weight w_{GT}. b) The absolute allele frequency of the composite genotype $Z_w = w_G G + w_{GT} GT$. Without loss of generality, here we assume $w_{GT} \geq 0$ and p = 0.5.

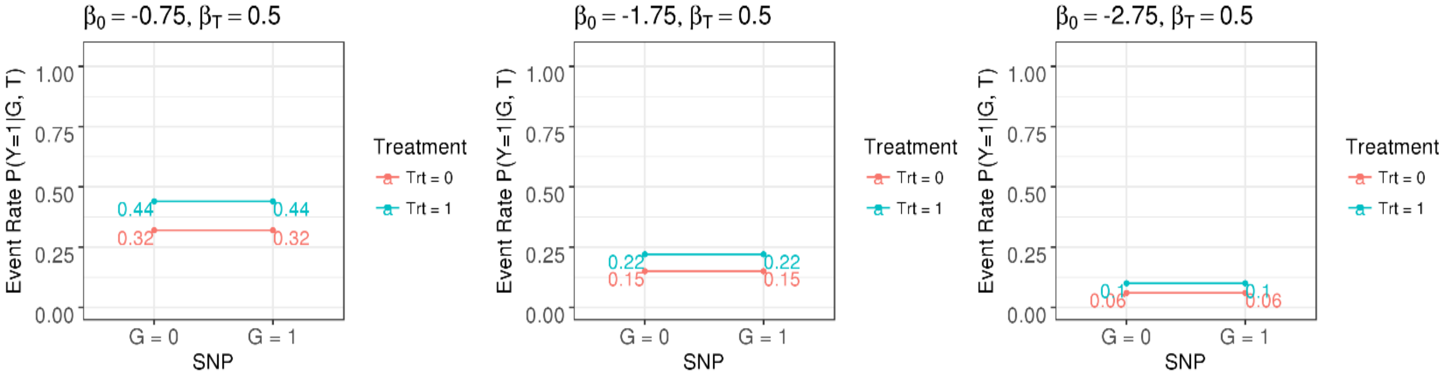

Figure S3. The event rate P(Y=1| G, T) corresponding to the three intercepts β_0, set as -0.75, -1.75 and -2.75, respectively. $\beta_T = 0.5$. No genotype by treatment interaction effect is assumed.

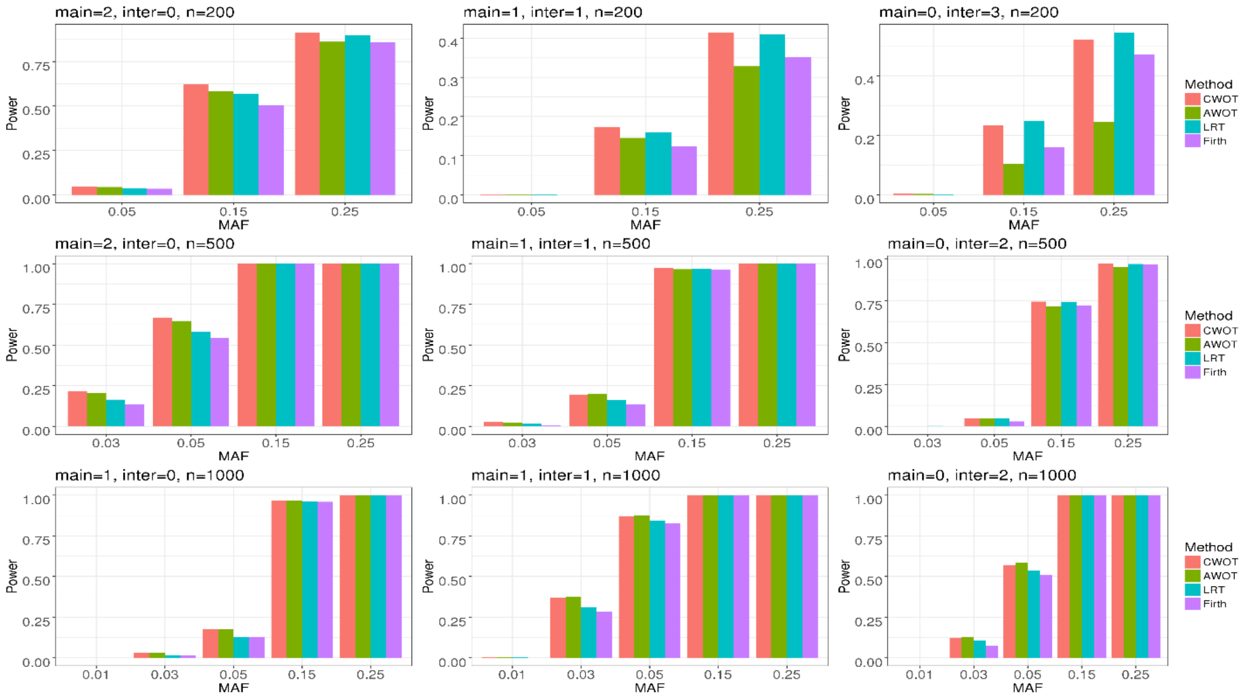

Figure S4. Power comparison of LRT, FT, AWOT, and CWOT when β_0 is set to -0.75 and the power is evaluated at $\alpha = 5 \times 10^{-8}$ for 2df tests of binary traits. "main" denotes the main G effect size β_G, inter denotes the G*T interaction effect size β_{GT} and n is sample size. MAF: minor allele frequency.

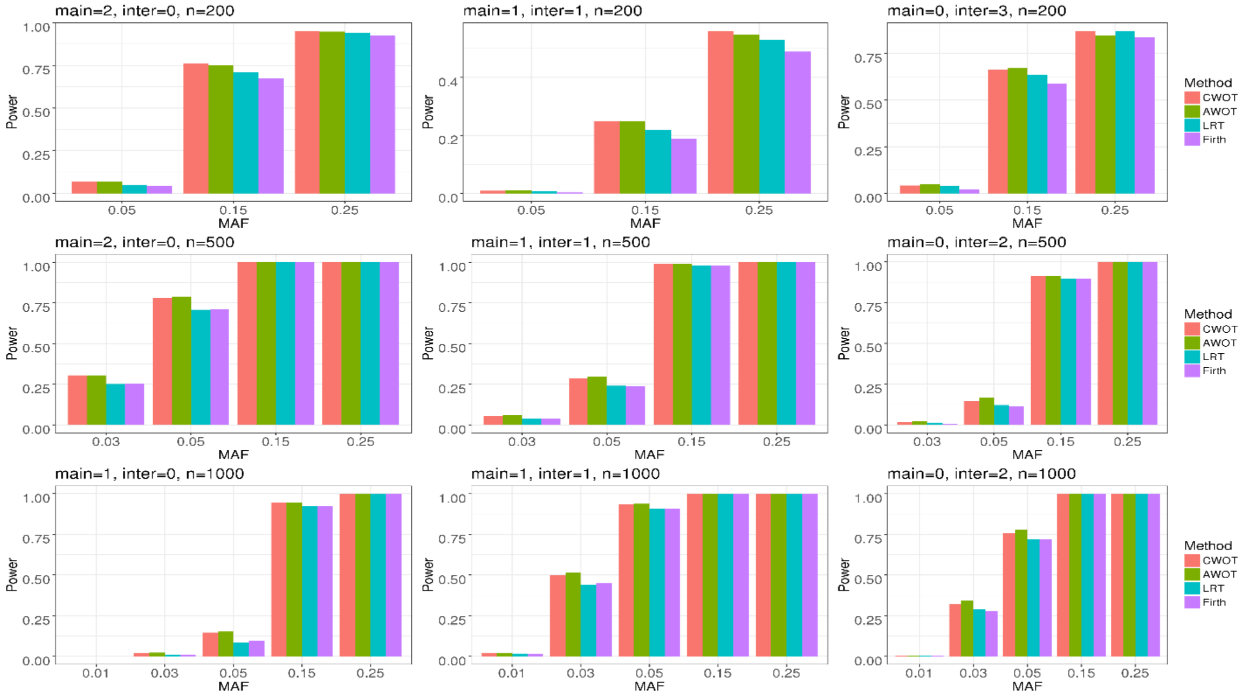

Figure S5. Power comparison of LRT, FT, AWOT and CWOT while β_0 is set as -1.75 and the power is evaluated at $\alpha = 5 \times 10^{-8}$ for 2df tests of binary traits. "main" denotes the main G effect size β_G, inter denotes the G*T interaction effect size β_{GT} and n is sample size. MAF: minor allele frequency.

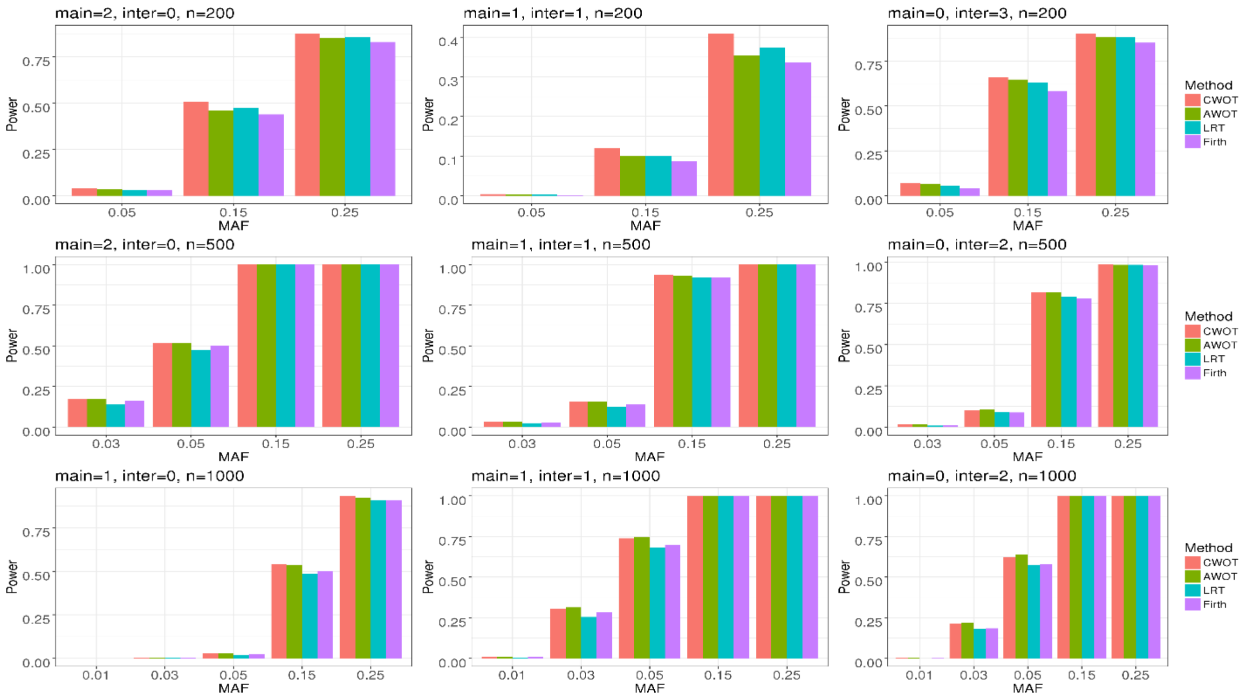

Figure S6. Power comparison of LRT, FT, AWOT and CWOT while β_0 is set as -2.75 and the power is evaluated at $\alpha = 5 \times 10^{-8}$ for 2df tests of binary traits. "main" denotes the main G effect size β_G, inter denotes the G*T interaction effect size β_{GT} and n is sample size. MAF: minor allele frequency.

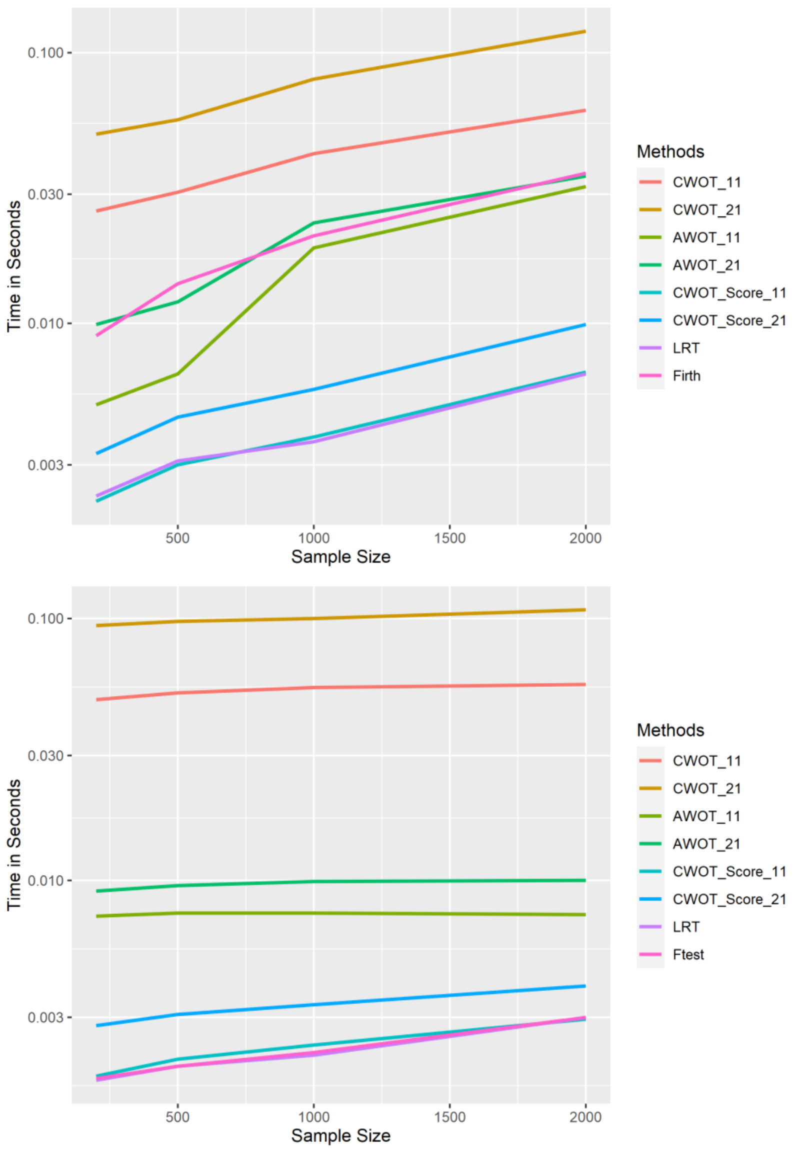

Figure S7. Computation time comparison between AWOT/CWOT and existing methods. Upper panel: binary trait. Lower panel: continuous trait. Simulation follows the Type I error simulation (Section 2.5.1). For each sample size, an average runtime of 500 repetitions is reported. The computation was conducted on a single core of 2.9 GHz Intel Core i7. CWOT_11 is the CWOT method with 11 equal-step grids from -1 to 1. CWOT_21 is the CWOT method with 21 equal-step grids from -1 to 1. AWOT, CWOT_Score methods follow the same setting. The maximum runtime shown above is around 0.125 second, which is equivalent to 34.72 CPU hours for GWAS of 1 million SNPs.

(a) LRT

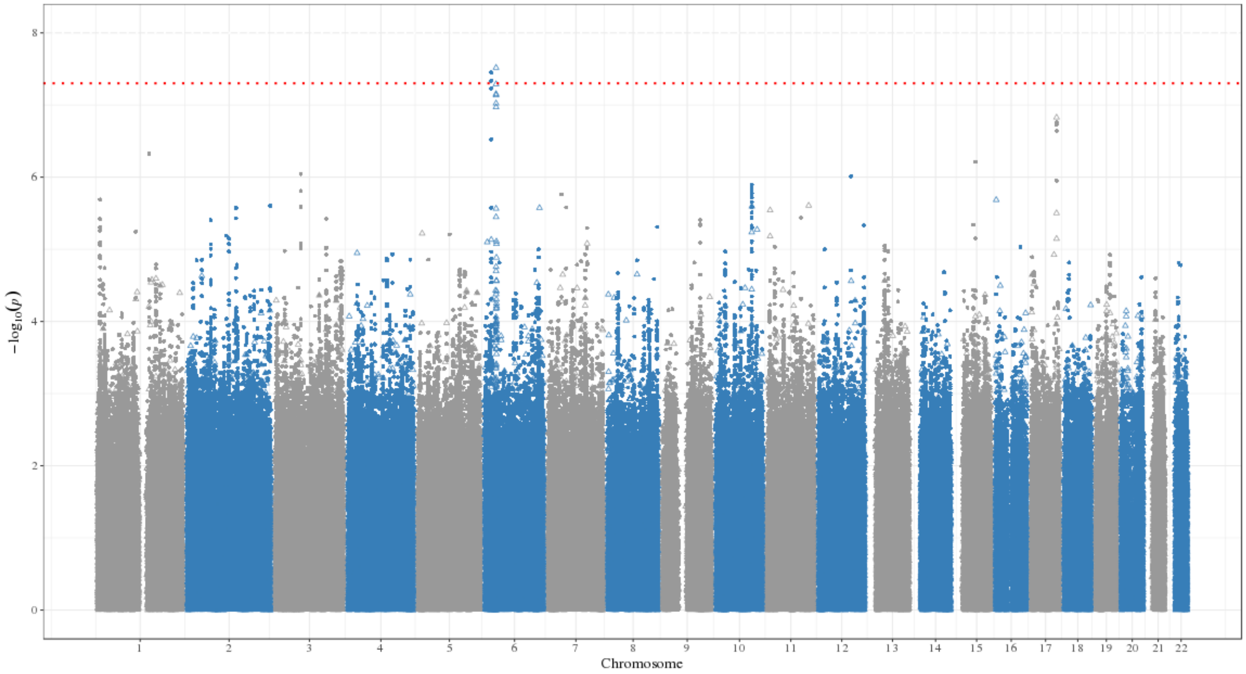
$-\log_{10}(p)$
Chromosome
0
2
4
6
8
1
2
3
4
5
6
7
8
9
10
11
12
13
14
15
16
17
18
19
20
21
22

(b) Firth

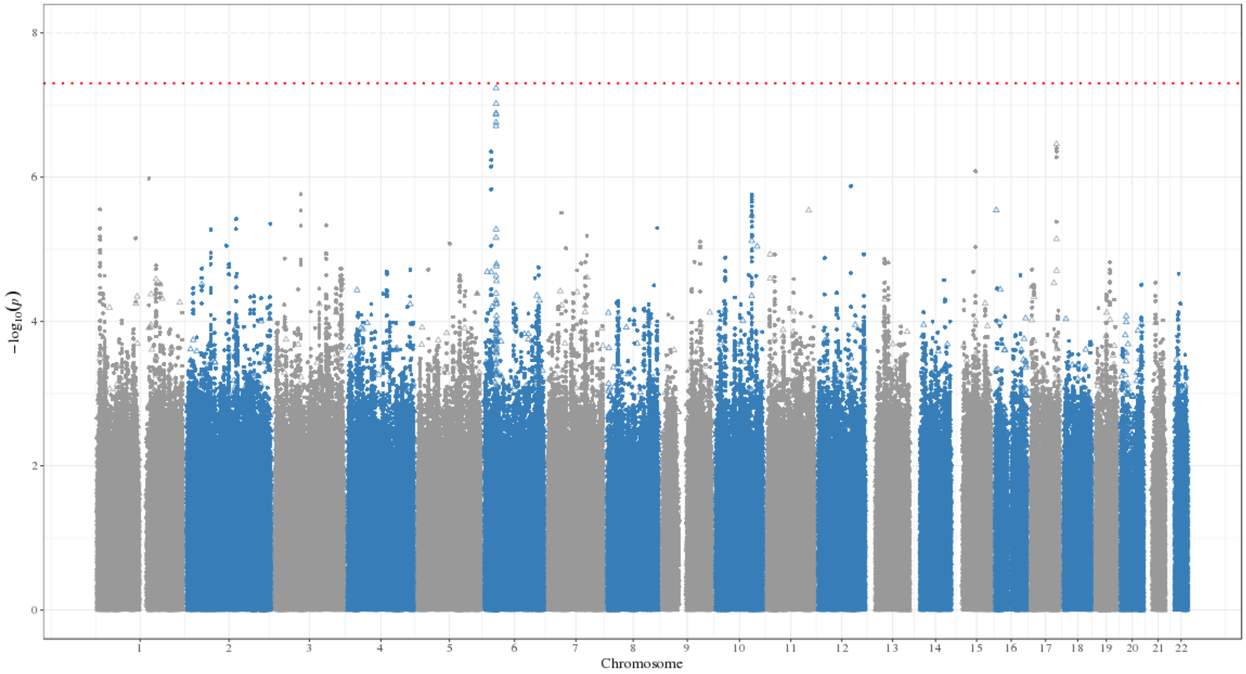
$-\log_{10}(p)$
Chromosome
0
2
4
6
8
1
2
3
4
5
6
7
8
9
10
11
12
13
14
15
16
17
18
19
20
21
22

(c) AWOT

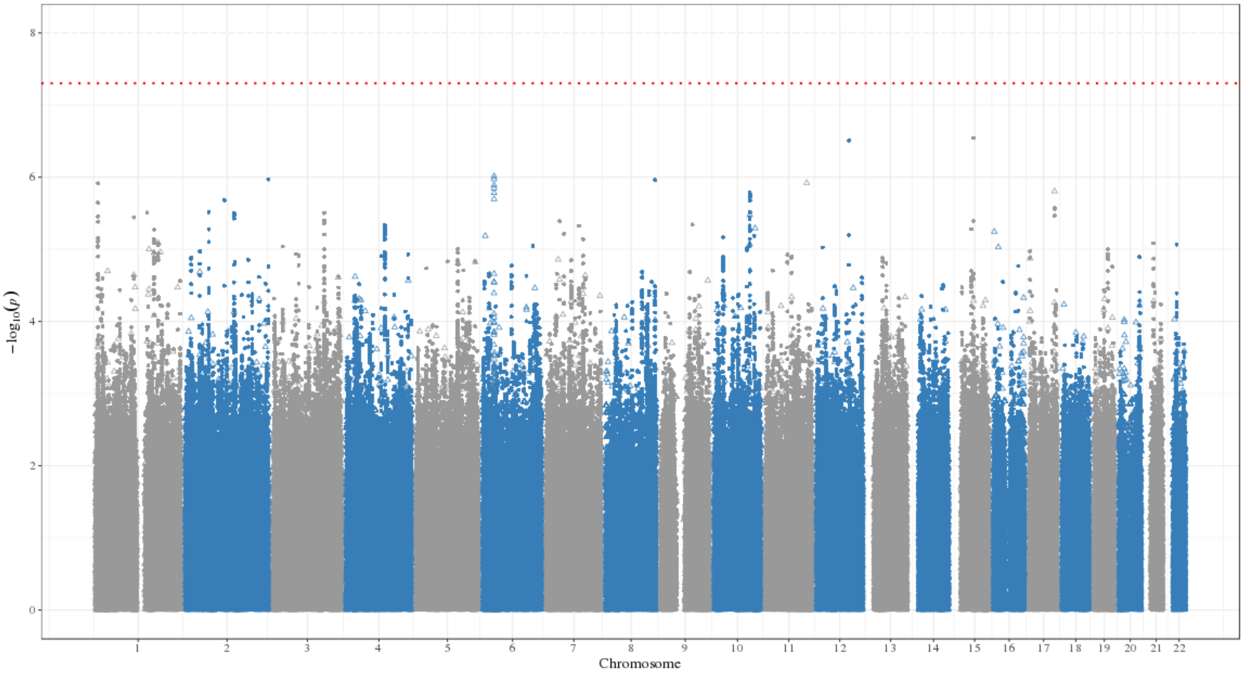

(d) CWOT_ESW

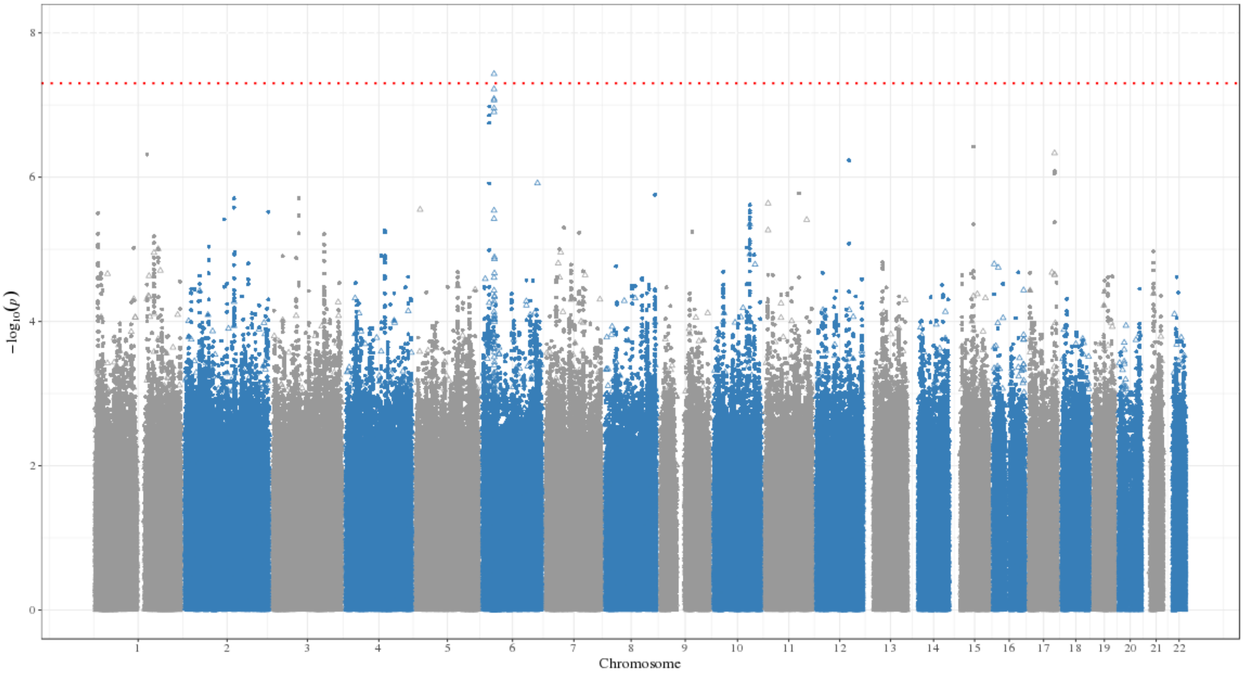

(e) CWOT_uESW01

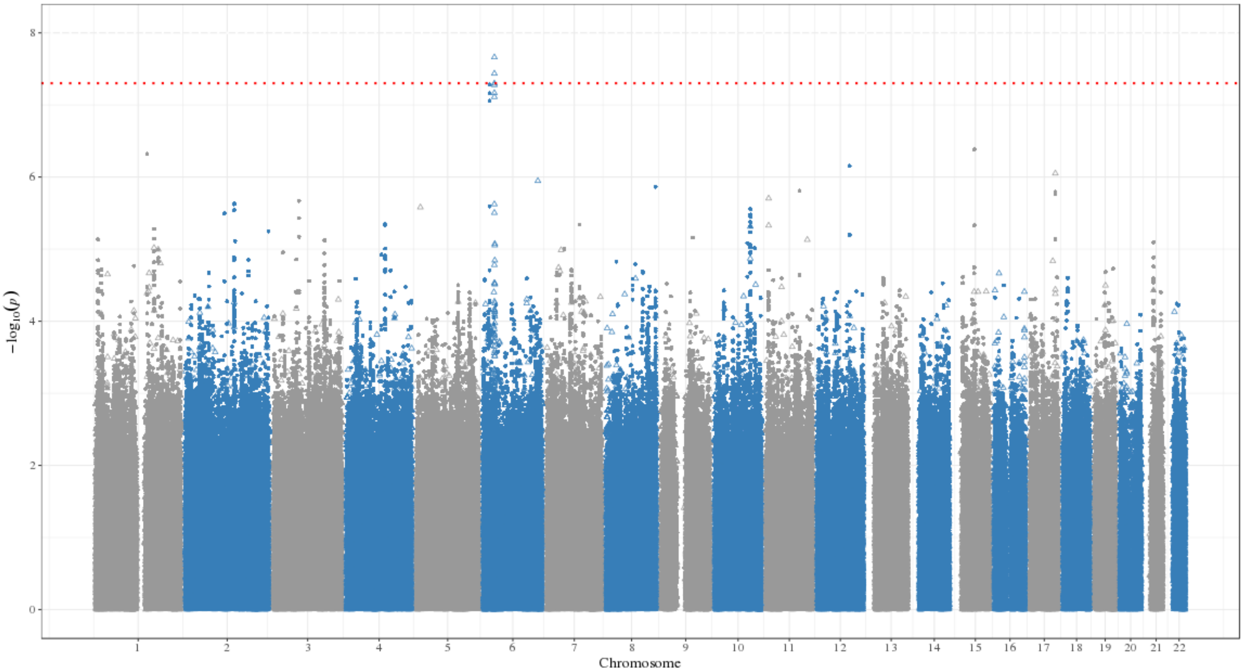

(f) CWOT_uESW0

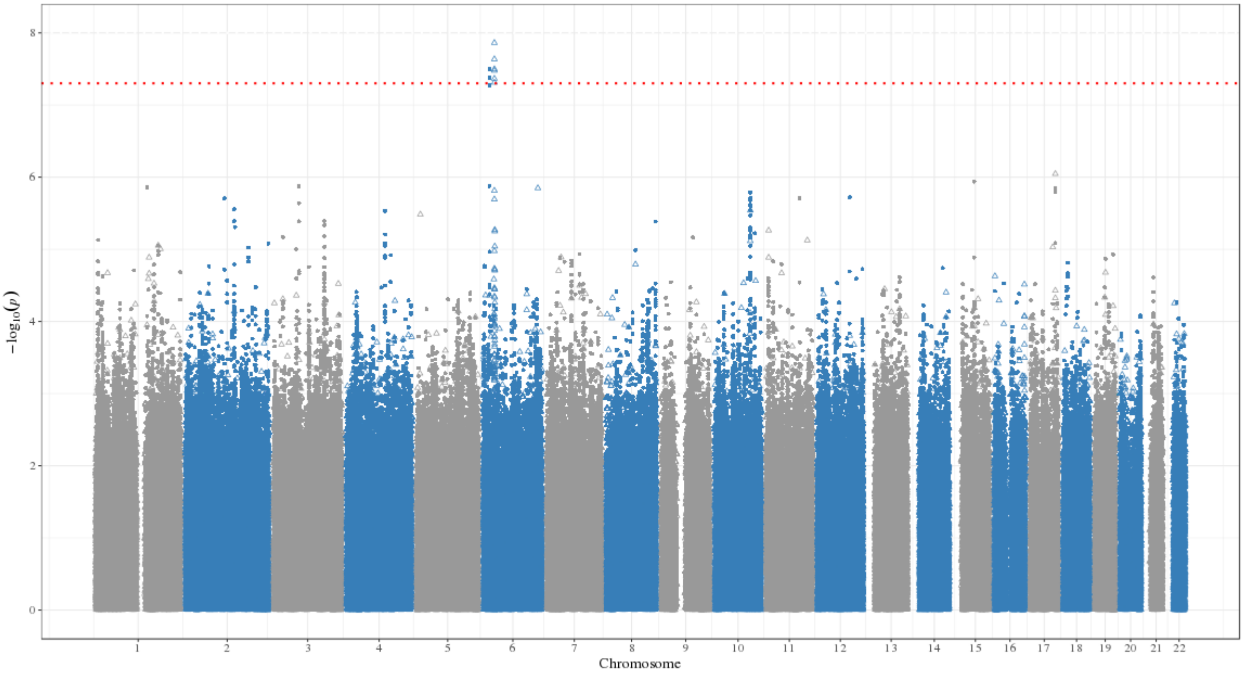

Figure S8. Manhattan plots of GWAS genotype and genotype by treatment interaction joint analysis of drug-induced reduction on rCDI (placebo arm versus bezlotoxumab and bezlotoxumab + actoxumab arms) from the LRT, Firth, AWOT, CWOT_ESW, CWOT_uESW01 and CWOT_uESW0 methods. The red dotted line is the genome-wide significance P value threshold of 5×10^{-8}. GWAS, genome-wide association study; rCDI, recurrent *Clostridium difficile* infection; SNP, single nucleotide polymorphism.

AWOT (minP_Score_SPA). CWOT_ESW (equal space weight: -1, -0.9, …, 0.9, 1). CWOT_uESW01 (weight more around 0, 1). CWOT_uESW0 (weight more around 0 only).

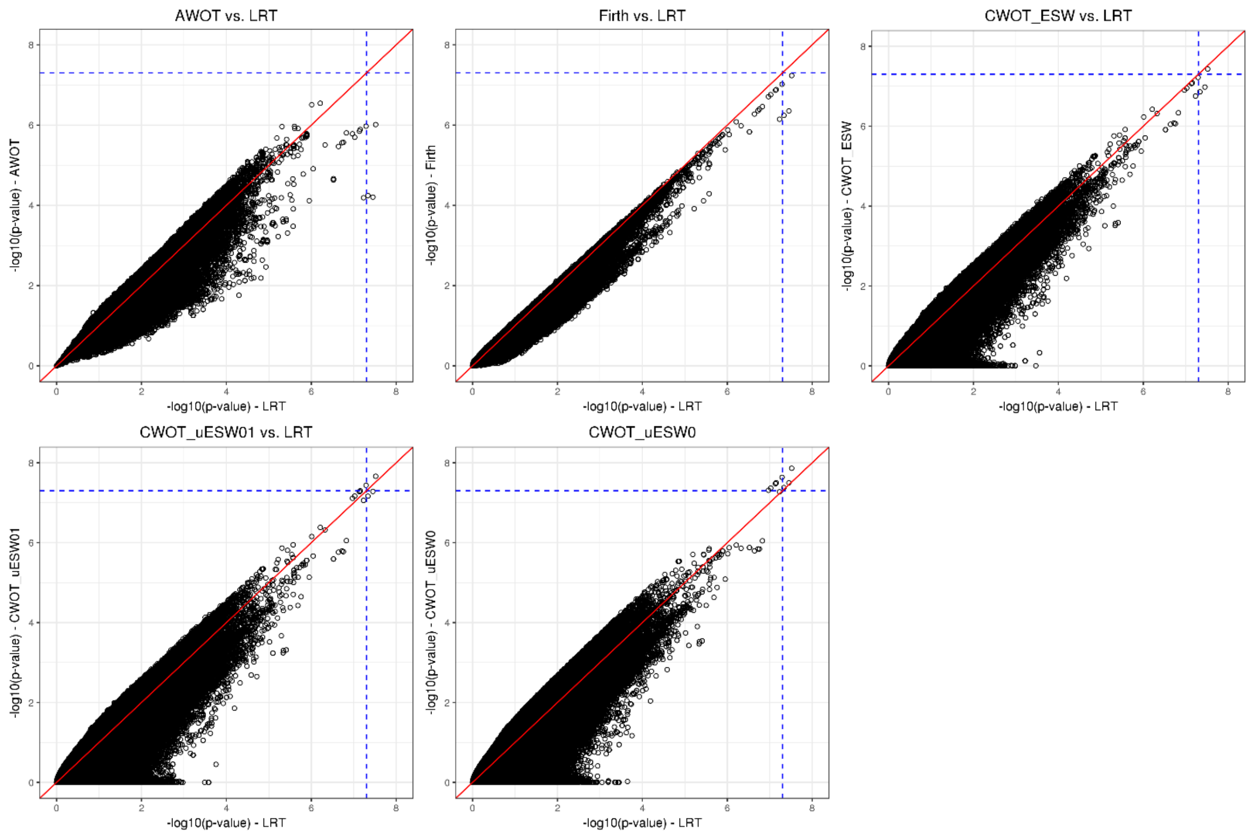

Figure S9. P-value comparison between Firth, AWOT, CWOT_ESW, CWOT_uESW01 and CWOT_uESW0 and LRT from the GWAS genotype and genotype by treatment interaction joint analysis of drug-induced reduction on rCDI (placebo arm versus bezlotoxumab and bezlotoxumab + actoxumab arms). The blue dotted line is the genome-wide significance P value threshold of 5×10^{-8}. GWAS, genome-wide association study; rCDI, recurrent *Clostridium difficile* infection; SNP, single nucleotide polymorphism. AWOT (minP_Score_SPA). CWOT_ESW (equal space weight: -1, -0.9, …, 0.9, 1). CWOT_uESW01 (weight more around 0, 1). CWOT_uESW0 (weight more around 0 only).

Table S1. Empirical type I error rates evaluated at $\alpha = 1 \times 10^{-5}$ for 2df test of binary trait. β_0 is set as -0.75, -1.75 and -2.75 respectively, $\beta_T = 0.5$, number of simulations: 5×10^6. FT: F test. Type I error ≥ 1.3α (3*SE) is marked in bold.

		$\beta_0 = -0.75$				$\beta_0 = -1.75$				$\beta_0 = -2.75$			
MAF	n	AWOT	CWOT	LRT	Firth	AWOT	CWOT	LRT	Firth	AWOT	CWOT	LRT	Firth
0.05	200	9.60E-06	1.21E-05	**1.33E-05**	1.40E-06	9.20E-06	8.80E-06	8.00E-06	5.20E-06	6.60E-06	6.10E-06	6.30E-06	5.00E-06
0.15	200	9.80E-06	**1.40E-05**	**1.59E-05**	6.80E-06	9.20E-06	8.60E-06	1.21E-05	6.20E-06	3.80E-06	6.97E-06	1.06E-05	5.60E-06
0.25	200	9.00E-06	1.09E-05	**1.32E-05**	9.00E-06	6.60E-06	1.12E-05	**1.61E-05**	7.20E-06	2.20E-06	1.02E-05	**1.56E-05**	4.60E-06
0.03	500	1.10E-05	1.26E-05	**1.43E-05**	5.00E-06	1.18E-05	8.50E-06	6.90E-06	4.60E-06	7.20E-06	5.35E-06	4.24E-06	7.40E-06
0.05	500	1.22E-05	1.19E-05	**1.40E-05**	5.60E-06	1.04E-05	1.06E-05	1.04E-05	5.40E-06	7.20E-06	5.40E-06	5.20E-06	6.00E-06
0.15	500	1.14E-05	1.00E-05	1.12E-05	1.06E-05	7.80E-06	1.05E-05	1.17E-05	8.20E-06	7.20E-06	8.60E-06	1.17E-05	7.40E-06
0.25	500	1.16E-05	1.02E-05	1.00E-05	8.80E-06	5.20E-06	9.90E-06	**1.30E-05**	8.20E-06	4.20E-06	8.59E-06	**1.52E-05**	9.60E-06
0.01	1000	7.20E-06	8.90E-06	1.01E-05	1.80E-06	8.40E-06	8.60E-06	7.80E-06	3.40E-06	8.80E-06	8.80E-06	6.20E-06	4.80E-06
0.03	1000	1.28E-05	1.28E-05	**1.59E-05**	8.00E-06	1.04E-05	1.10E-05	1.10E-05	6.40E-06	9.40E-06	8.38E-06	6.16E-06	7.00E-06
0.05	1000	1.10E-05	1.10E-05	1.29E-05	8.00E-06	1.00E-05	1.08E-05	**1.44E-05**	6.80E-06	8.00E-06	7.82E-06	8.73E-06	5.00E-06
0.15	1000	1.22E-05	1.14E-05	1.17E-05	8.00E-06	8.40E-06	1.08E-05	1.22E-05	9.60E-06	8.20E-06	9.35E-06	**1.38E-05**	6.20E-06
0.25	1000	**1.38E-05**	9.30E-06	1.10E-05	1.20E-05	1.02E-05	9.50E-06	1.11E-05	1.00E-05	4.80E-06	7.41E-06	9.34E-06	7.40E-06

Table S2. Association results of the GWAS genotype and genotype by treatment interaction joint analysis of drug-induced reduction on rCDI (placebo arm versus bezlotoxumab and bezlotoxumab + actoxumab arms) from the LRT, Firth, AWOT and CWOT_ESW, CWOT_uESW01 and CWOT_uESW0 methods. The p-values passing the genome-wide significance threshold 5×10^{-8} are highlighted in bold. ".p" denotes p-value and ".w" denotes the optimal weight which yields the minimal p-value.

SNP	CHR	POS	MAF	LRT.p	Firth.p	AWOT.p	CWOT_ESW.p	CWOT_uESW01.p	CWOT_uESW0.p	AWOT.w	CWOT_ESW.w	CWOT_uESW01.w	CWOT_uESW0.w
rs2516513	6	31447588	0.23	**3.04E-08**	5.84E-08	9.70E-07	**3.71E-08**	**2.18E-08**	**1.37E-08**	0	0	0.004	0.008
rs2516509	6	31449994	0.24	5.14E-08	9.62E-08	1.06E-06	6.03E-08	**3.67E-08**	**2.31E-08**	0	0	0.032	0.027
rs113379306	6	17333351	0.04	**3.54E-08**	4.44E-07	6.24E-05	1.05E-07	5.21E-08	**3.17E-08**	-0.5	-0.25	-0.032	-0.027
rs2523705	6	31451680	0.23	7.02E-08	1.30E-07	1.29E-06	8.23E-08	5.03E-08	**3.18E-08**	0	0	0.032	0.027
rs2248462	6	31446796	0.23	7.33E-08	1.36E-07	1.42E-06	8.58E-08	5.28E-08	**3.33E-08**	0.25	0	0.032	0.027
rs76166871	6	17329940	0.04	**4.64E-08**	5.74E-07	5.69E-05	1.38E-07	6.88E-08	**4.19E-08**	-0.5	-0.25	-0.032	-0.027
rs2516511	6	31448625	0.24	9.49E-08	1.75E-07	2.01E-06	1.12E-07	6.83E-08	**4.31E-08**	0	0	0.004	0.008
rs2516422	6	31449269	0.23	1.07E-07	1.96E-07	1.65E-06	1.25E-07	7.73E-08	**4.88E-08**	0.25	0	0.032	0.027
